# FTO promotes tumour proliferation in bladder cancer via the FTO/miR-576/CDK6 axis in an m6A-dependent manner

**DOI:** 10.1038/s41420-021-00724-5

**Published:** 2021-11-01

**Authors:** Guanwen Zhou, Keqiang Yan, Jikai Liu, Lijian Gao, Xianzhou Jiang, Yidong Fan

**Affiliations:** 1 Department of Urology, Qilu Hospital, Cheeloo College of Medicine, Shandong University, Jinan, China; 2 Department of Urology, Dezhou People’s Hospital, Dezhou, China

**Keywords:** miRNAs, Bladder cancer, Urological cancer, Methylation

## Abstract

The aberrant expression of fat mass and obesity-associated protein (FTO) has been confirmed to be associated with a variety of cancers and participates in the regulation of multiple biological behaviours. FTO plays an oncogenic role in bladder cancer, but few studies have focused on how FTO promotes bladder cancer progression by regulating miRNA synthesis. Here, we confirmed that FTO expression was significantly increased in bladder cancer and was associated with a poor prognosis. FTO overexpression promoted bladder cancer cell proliferation, whereas FTO knockdown inhibited bladder cancer cell proliferation. We also demonstrated that FTO promoted bladder cancer cell proliferation via the FTO/miR-576/CDK6 pathways. Taken together, our work revealed that FTO plays a critical role in bladder cancer and could be a potential diagnostic or prognostic biomarker for this disease.

## Introduction

Bladder cancer is a complex disease associated with high morbidity and mortality rates if not optimally treated [1]. The occurrence of bladder cancer is a complicated, multifactor, and multistep pathological process and is affected by both internal genetic factors and external environmental factors [2]. Although surgical resection is a conventional and effective clinical treatment for non-muscle-invasive bladder cancer (NMIBC), the latter still recurs and metastasises easily. Therefore, the study of the underlying molecular mechanisms of bladder cancer and the discovery of new therapeutic targets thereof has great significance.

RNA methylation modifications account for more than 60% of all RNA modifications, and N6-methyladenine(m6A) is one of the most common modifications on the mRNA and ncRNA levels [3]. The aberrant regulation of this modification is closely associated with the occurrence and development of many malignancies [4]. The fat mass and obesity-associated (FTO) gene is a common m6A demethylase that has been proven to have cancer-promoting activity in gastric cancer, breast cancer, bladder cancer and cervical squamous cell carcinoma [5–10]. Some studies have shown that FTO affects tumour progression via regulating microRNA (miRNA) expression [11, 12].

MicroRNAs(miRNAs) are responsible for the regulation of gene expression at the post-transcriptional modification manner by targeting mRNAs and forming RNA-induced silencing complexes (RISC), thereby regulating cell growth, proliferation, differentiation and apoptosis. Many researches have verified that m6A could regulate the formation of mRNA and non-coding RNA in a post-transcriptional modification manner [13–15].

The present study demonstrated that FTO exhibited an oncogenic role in bladder cancer via regulating the expression of cyclin-dependent kinases (CDK6), which are closely related to the cell cycle. Patients with bladder cancer exhibiting high FTO levels may have a worse prognosis. This indicates that FTO exhibits an oncogenic role via the FTO/miR-576/CDK6 pathways in bladder cancer and could be a potential diagnostic or prognostic biomarker for bladder cancer.

## Result

### FTO was obviously upregulated in bladder cancer tissues and cell lines and closely associated with clinicopathological parameters

**Fig. 1:**
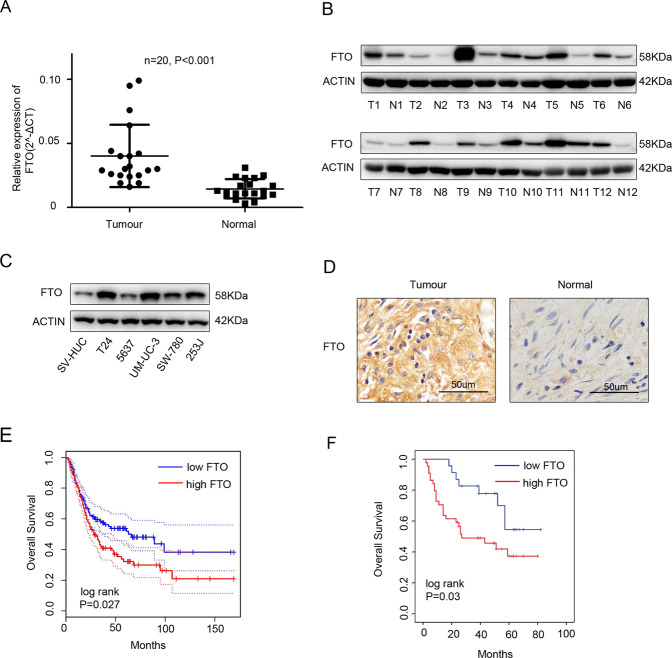
FTO was upregulated in bladder cancer tissues and cell lines and closely associated with clinicopathological parameters. **A** qRT-PCR analysis of FTO mRNA expression in 20 paired bladder cancer tissues (Tumour) and matched adjacent normal tissues (Normal). **B** The expression of FTO proteins in bladder cancer tissues (T) and adjacent normal tissues (N) using western blotting. **C** The expression of FTO proteins in SV-HUC cell lines and in bladder cancer cell lines using western blotting. **D** IHC analysis of FTO protein expression in bladder cancer tissues (Tumour) and matched adjacent normal tissues (Normal) at a 400× magnification. Scale bars represent 50 μm. **E**, **F**. Kaplan–Meier survival curves of overall survival (OS) based on Gepia database and tissue microarray data. Between-group differences were compared using log-rank test (*P* = 0.027 and 0.03, respectively).

We initially detected FTO expression in bladder cancer tissues and adjacent tissues using qRT-PCR and western blotting. The expression levels of FTO were significantly higher in bladder cancer tissues (Fig. 1A, B). Moreover, we also analysed FTO expression in T24, 5637, UM-UC-3, SW 780, 253 J and SV-HUC cell lines and found that the mRNA and protein levels of FTO were significantly higher in bladder cancer cell lines than in the normal urinary epithelial cell line SV-HUC (Fig. 1C). The IHC analysis showed that the positive FTO rate was significantly higher in bladder cancer tissues (Fig. 1D). The tissue microarray analysis revealed that the expression of FTO was correlated with the TNM stage. The T stage was higher in the FTO group with high expression (Table 1). We predicted the prognosis of FTO in bladder cancer using the Gepia database (http://gepia.cancer-pku.cn/) and found that patients with bladder cancer exhibiting high FTO levels may have a worse prognosis (Fig. 1E). Moreover, the same conclusion was drawn from the tissue microarray data (Fig. 1F).

**Table 1:** Correlation between FTO expression and clinicopathological characteristics of 67 bladder cancer patients.

Parameters	Number of cases	FTO expression^a^		P Value
		Low	High	
**All cases**	67	23	44	
**Age(years)**				
<65	24	8	16	0.995
≥65	43	15	28	
**Gender**				
Male	57	20	37	0.755
Female	10	3	7	
**TNM stage**				
pTa-pT1	21	11	10	0.035*
pT2-pT4	46	12	34	
**Histological grade**				
Low	9	5	4	0.14
high	58	18	40	

*Statistically significant, *P* < 0.05

^a^On the basis of the IHC scores, the patients were divided into low (0–3) and high (4–9) FTO groups. Final IHC score = percentage of positive cells × intensity score.

### The overexpression of FTO promoted bladder cancer cell proliferation and invasion in vitro and in vivo

**Fig. 2:**
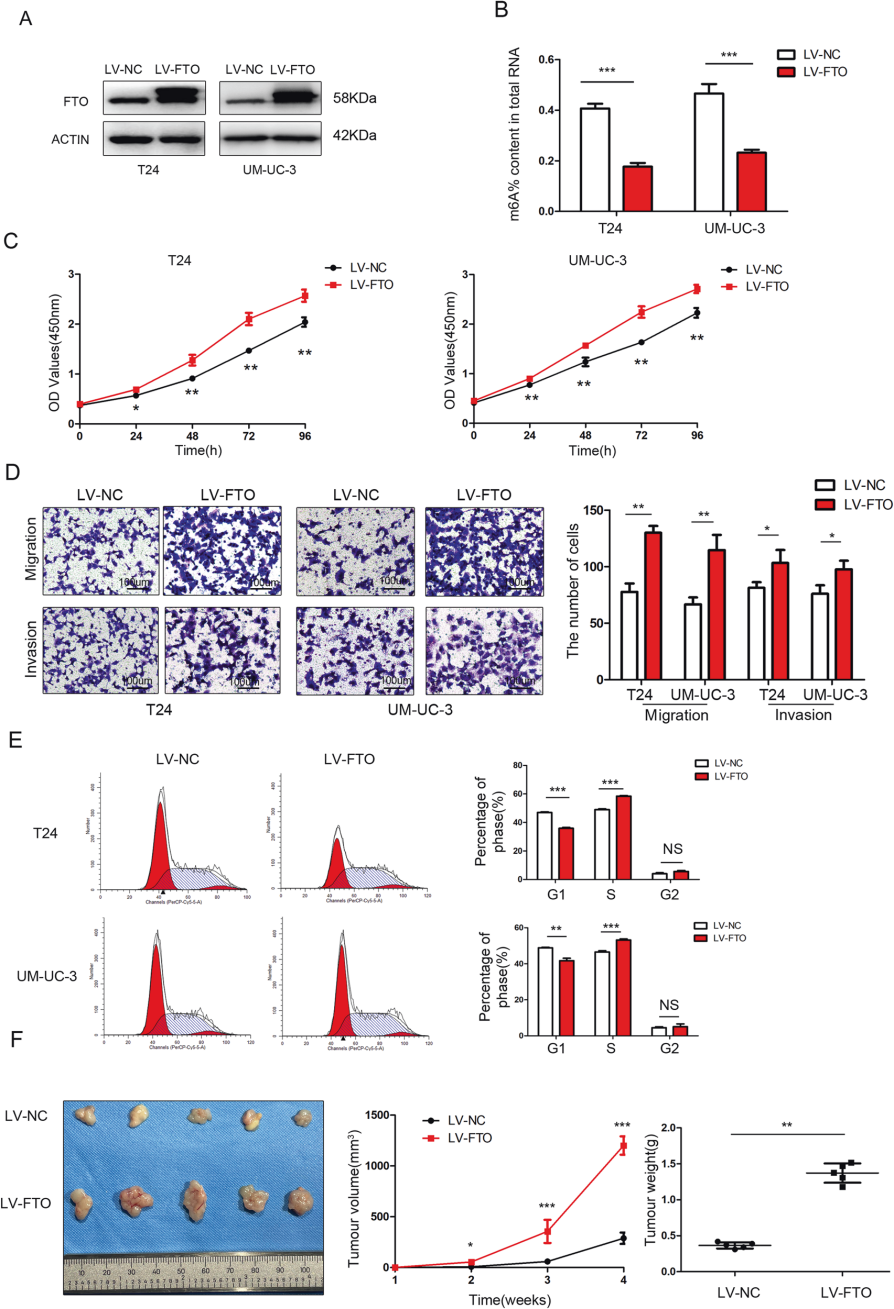
The overexpression of FTO promoted bladder cancer cell proliferation and invasion in vitro and in vivo. **A** FTO was detected by western blotting after FTO overexpression in T24 and UM-UC-3 cells. **B** The m6A levels were measured in FTO-overexpressing T24 and UM-UC-3 cells. **C** FTO overexpression promoted cell proliferation determined by CCK-8 assays. **D** Transwell migration and invasion assays after FTO overexpression at a 100× magnification. Scale bars represent 100 μm. **E** Cell-cycle analysis was performed in FTO-overexpressing T24 and UM-UC-3 cells using flow cytometry. **F** Image of tumours collected from nude mice. The tumour growth curve and tumour weight were measured in the FTO-overexpression group and the control group. The results are presented as mean ± standard deviation (SD). *P < 0.05, **P < 0.01, ***P < 0.001.

FTO expression was confirmed by western blotting in stably transfected T24, 5637 and UM-UC-3 cell lines (Fig. 2A and Supplementary Fig. 1A). Owing to the m6A demethylase function of FTO, the relative m6A level decreased after the overexpression of FTO (Fig. 2B and Supplementary Fig. 1B).

The results of the CCK-8 assay showed that the proliferation of T24 and UM-UC-3 cells increased significantly after the overexpression of FTO (Fig. 2C). The migration and invasion abilities of T24 and UM-UC-3 cells significantly increased after the overexpression of FTO (Fig. 2D). Flow cytometry analysis showed a decrease in the percentage of FTO-overexpressing cells in the G0/G1 phase and an increase in the percentage in the S phase (Fig. 2E). The above assays were conducted in stably transfected 5637 cell lines as well (Supplementary Fig. 1 C–E) .

In the subcutaneous xenograft tumour model, the growth of tumours injected with FTO-overexpressing T24 cells was obviously promoted compared with those injected with control cells (Fig. 2F).

### FTO knockdown inhibited bladder cancer cell proliferation and invasion in vitro and in vivo

**Fig. 3:**
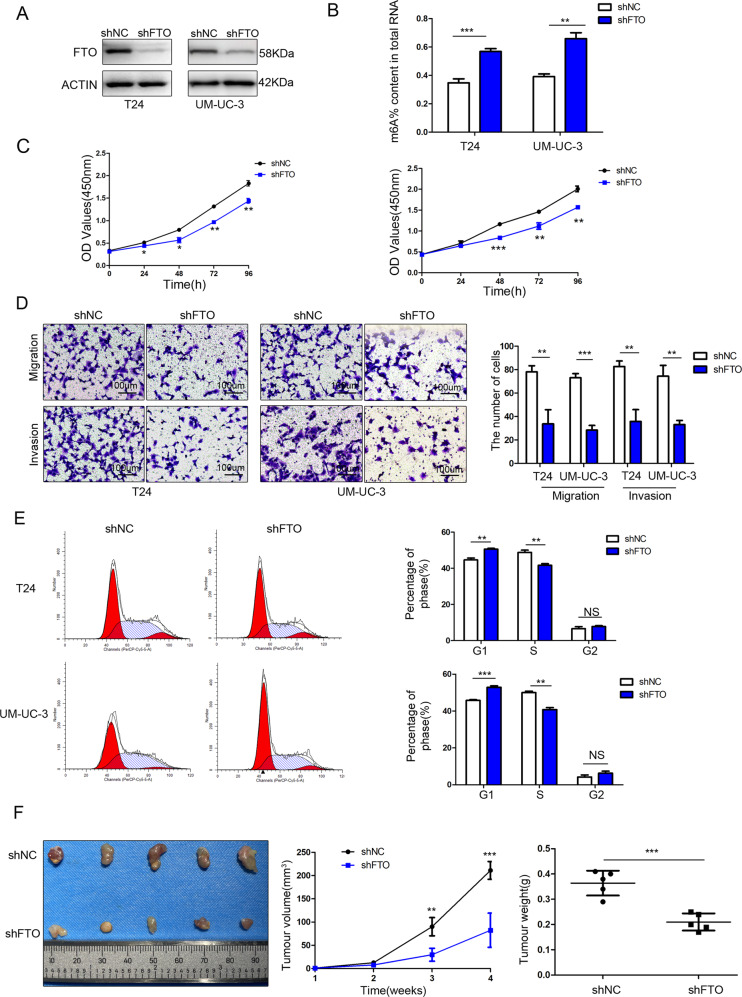
FTO knockdown inhibited bladder cancer cell proliferation and invasion in vitro and in vivo. **A** FTO was detected by western blotting after FTO knockdown in T24 and UM-UC-3 cells. **B** The m6A levels were measured in FTO-knockdown T24 and UM-UC-3 cells. **C** CCK-8 assay showed that FTO knockdown inhibited cell proliferation. **D** Transwell migration and invasion assays after FTO knockdown at a 100× magnification. Scale bars represent 100 μm. **E** Cell-cycle analysis was performed in FTO-knockdown T24 and UM-UC-3 cells using flow cytometry. **F** Image of tumours collected from nude mice. The tumour growth curve and tumour weight were measured in the FTO knockdown group and the control group. The results are presented as mean ± standard deviation (SD). *P < 0.05, **P < 0.01, ***P < 0.001.

FTO expression was confirmed by western blotting in stable FTO-knockdown T24 and UM-UC-3 cells (Fig. 3A). The relative m6A level increased after FTO knockdown (Fig. 3B).

CCK-8 assays showed that cell proliferation decreased significantly after FTO knockdown (Fig. 3C). The migration and invasion abilities of T24 and UM-UC-3 cells decreased significantly after FTO knockdown (Fig. 3D). Flow cytometry analysis showed that the percentage of cells in the G0/G1 phase increased and that of the S phase decreased in FTO knockdown cells (Fig. 3E).

In the subcutaneous xenograft tumour model, the growth of tumours injected with FTO knockdown T24 cells was obviously inhibited (Fig. 3F).

### FTO regulated the processing of miR-576

**Fig. 4:**
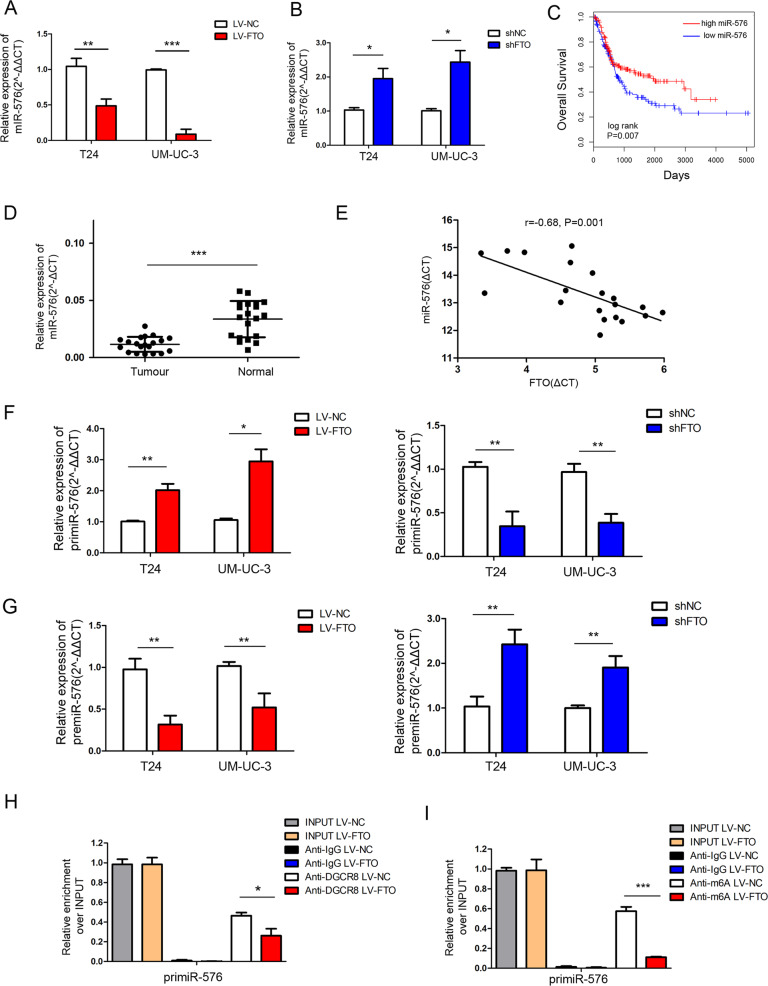
FTO regulated the processing of miR-576. **A**, **B** The expression of miR-576 was determined by qRT-PCR after FTO overexpression or knockdown. **C** Kaplan–Meier analysis of overall survival based on the LinkedOmics database. Between-group differences were compared using a log-rank test. (*P* = 0.007). **D** miR-576 expression in 20 paired bladder cancer tissues (Tumour) and adjacent normal tissues (Normal) by qRT-PCR. **E** Correlation analysis was performed between FTO and miR-576 expressions (*r* = −0.68, *P* = 0.001) in 20 tumour tissues. **F**, **G** The relative expression of primiR-576 and premiR-576 was detected by qRT-PCR. **H** qRT-PCR analysis of primiRNA binding to DGCR8 via RNA immunoprecipitation from FTO-overexpressing T24 cells and negative control cells. **I** qRT-PCR analysis of primiRNA modified by m6A via m6A immunoprecipitation from FTO-overexpressing T24 cells and negative control cells. The results are presented as mean ± standard deviation (SD). *P < 0.05, **P < 0.01, ***P < 0.001.

Considering that reduced m6A levels led to the arrest of the primiRNA process [1, 14, 16], the expression of FTO-related miRNAs was negatively correlated with FTO expression. Using the LinkedOmics database [17] (http://www.linkedomics.org/login.php), we found that miR-19a, miR-17, miR-590, miR-93, miR-576 and miR-192 were negatively correlated with FTO in bladder cancer (Supplementary Fig. 2). We verified that only miR-576 was altered in T24 and UM-UC-3 cells after FTO overexpression or knockdown by qRT-PCR (Fig. 4A, B). By the LinkedOmics database [17] (http://www.linkedomics.org/login.php), we discovered that the low expression of miR-576 was associated with poor overall survival in bladder cancer patients (Fig. 4C).

We detected miR-576 expression in 20 pairs of bladder cancer tissue samples using qRT-PCR and found that the miR-576 expression was significantly decreased in bladder cancer tissues (Fig. 4D). Moreover, a negative correlation between the expression of FTO and miR-576 (*r* = −0.68, *P* = 0.001) was revealed in 20 tumour tissues (Fig. 4E). Additionally, unprocessed primiRNA-576 was found to accumulate in FTO-overexpressing cells and accelerate in FTO knockdown cells, while opposite results were obtained for the premiR-576 level (Fig. 4F, G).

DGCR8 was immunoprecipitated from FTO-overexpressing T24 cells and negative control cells. The expression levels of DGCR8-bound primiR-576 were significantly decreased in FTO-overexpressing T24 cells (Fig. 4H). Furthermore, m6A was immunoprecipitated from FTO-overexpressing T24 cells and negative control cells, revealing that the expression levels of primiR-576 modified by m6A significantly decreased in FTO-overexpressing T24 cells (Fig. 4I). These results indicate that FTO regulates the processing of miR-576 in an m6A-depend manner.

### miR-576 rescued the proliferating function induced by FTO

**Fig. 5:**
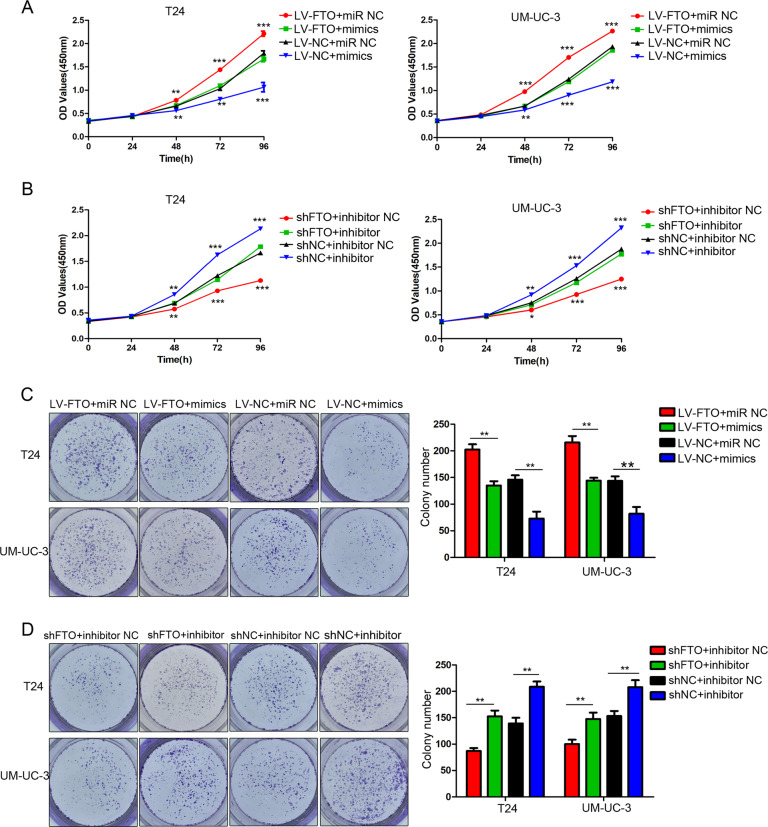
miR-576 rescued the FTO-induced proliferative function in bladder cancer cells. **A**, **B** The effect of miR-576 mimics or inhibitors on T24 and UM-UC-3 cells with FTO overexpression and knockdown was detected by CCK-8 assays. **C**, **D** The effect of miR-576 mimics or inhibitors on colony formation ability in T24 and UM-UC-3 cells with FTO overexpression and knockdown was determined by colony formation assays. The results are presented as mean ± standard deviation (SD). *P < 0.05, **P < 0.01, ***P < 0.001.

We upregulated miR-576 in stable FTO-overexpressing cells and performed a series of restoration assays. Our results showed that the overexpression of miR-576 reversed the effects of FTO overexpression on cell proliferation (Fig. 5A) and colony formation ability (Fig. 5C). We downregulated miR-576 in stable FTO-knockdown T24 and UM-UC-3 cells and demonstrated that the inhibition of miR-576 expression partly restored the decreased proliferation (Fig. 5B) and colony formation (Fig. 5D) abilities of bladder cancer cells.

### FTO targeted CDK6 via the FTO/miR-576/CDK6 pathways in bladder cancer

**Fig. 6:**
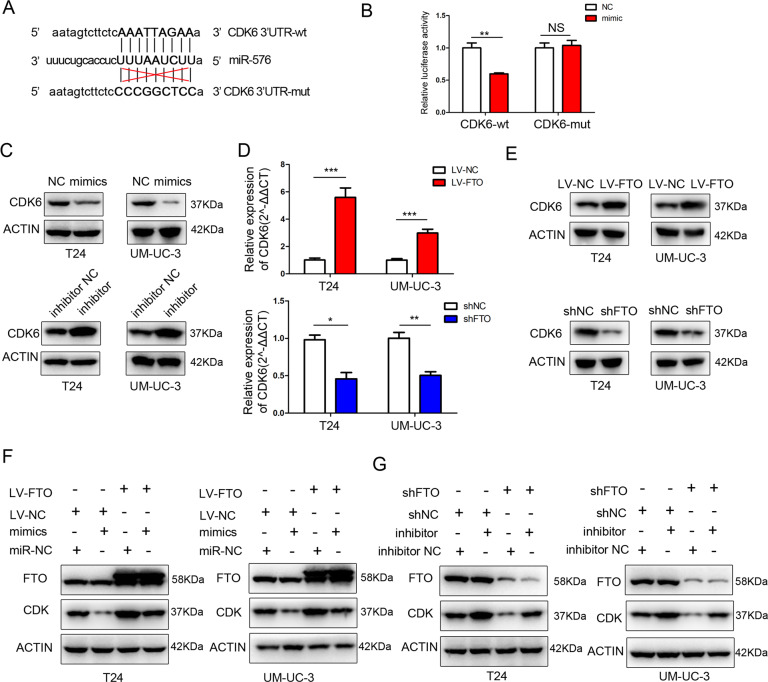
FTO targeted CDK6 via the FTO/miR-576/CDK6 pathways in bladder cancer. **A** miR-576 and its predicted binding sequence in the wild-type (wt) and mutant (mut) 3′-UTRs of CDK6 mRNA. **B** Luciferase assays validated that CDK6 is a target of miR-576. **C** The effect of miR-576 mimics or inhibitors on CDK6 expression was measured by western blotting. **D**, **E** The expression of CDK6 was determined by qRT-PCR and western blotting in T24 and UM-UC-3 cells after FTO overexpression or knockdown. **F**, **G** Western blotting showed that miR-576 could partially rescue the expression of CDK6 induced by FTO. The results are presented as mean ± standard deviation (SD). *P < 0.05, **P < 0.01, ***P < 0.001.

Potential target genes of miR-576 were predicted using databases such as TargetScan (http://www.targetscan.org) and miRDB (http://mirdb.org/) (Supplementary Table 1), we discovered binding sites for miR-576 in the 3′-untranslated regions (UTRs) of CDK6 mRNA (Fig. 6A). Next, we performed a dual-luciferase reporter assay to test whether CDK6 is a direct target of miR-576. The luciferase activity decreased significantly with miR-576 overexpression in the wild-type 3′-UTR sequence of CDK6 compared with the mutant 3′-UTR sequence of CDK6 (Fig. 6B), suggesting that CDK6 is a direct downstream target of miR-576. Additionally, the expression of CDK6 decreased after miR-576 mimics transfection and increased after miR-576 inhibitor transfection into bladder cancer cells (Fig. 6C). Moreover, the expression of CDK6 increased significantly in FTO-overexpressing bladder cancer cells and decreased significantly in FTO knockdown bladder cancer cells (Fig. 6D, E). Lastly, the transfection of FTO-overexpressing cells with miR-576 mimics partially reduced the expression of CDK6 induced by the overexpression of FTO. In contrast, the transfection of the miR-576 inhibitor into FTO knockdown cells partially reversed the inhibition of CDK6 expression by FTO downregulation (Fig. 6F, G). Taken together, these results indicate that FTO targets CDK6 via the FTO/miR-576/CDK6 pathways in bladder cancer.

### The expression of CDK6 was positively correlated with FTO in bladder cancer tissues

**Fig. 7:**
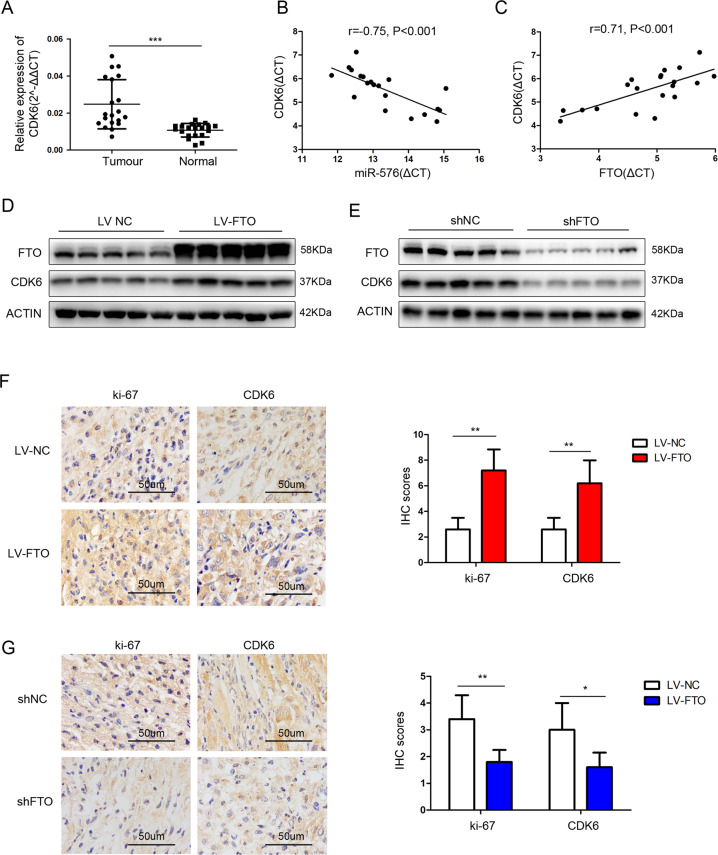
CDK6 was positively correlated with FTO expression in bladder cancer tissues. **A** CDK6 mRNA expression in 20 paired bladder cancer tissues (Tumour) and matched adjacent normal tissues (Normal) by qRT-PCR. **B** Correlation analysis between the expression of miR-576 and CDK6 (*r* = −0.75, *P* < 0.001) in 20 tumour tissues. **C** Correlation analysis between the expression of FTO and CDK6 (*r* = 0.71, *P* < 0.001) in 20 tumour tissues. **D**, **E** Proteins were extracted from the tumours collected from nude mice. The protein expression of FTO and CDK6 was measured by western blotting. **F**, **G** The expression of ki-67 and CDK6 in xenograft tumour tissues was measured by IHC. (Magnification, ×400, scale bars represent 50 μm). *P < 0.05, **P < 0.01, ***P < 0.001.

The expression of CDK6 increased significantly in bladder cancer tissues (Fig. 7A). A negative correlation was observed between miR-576 and CDK6 expressions (*r* = −0.75, *P* < 0.001) (Fig. 7B), whereas a positive correlation was observed between FTO and CDK6 expressions (*r* = 0.71, *P* < 0.001) (Fig. 7C) in 20 tumour tissues. FTO and CDK6 protein levels in mouse xenograft model tissues were measured by western blotting. The expression of CDK6 in the tumours injected with FTO-overexpressing cells increased significantly compared to those injected with control cells (NC), while opposite results were obtained in the FTO-knockdown group (Fig. 7D, E). The IHC of subcutaneous xenograft tumours showed that the positive rate of CDK6 and Ki-67 increased significantly in the FTO-overexpression group and decreased significantly in the FTO-knockdown group (Fig. 7F, G). These results indicate that CDK6 is positively correlated with FTO expression in bladder cancer tissues.

## Discussion

As one of the crucial enzymes of m6A modification, FTO played an indispensable role in obesity and cancer [5–10, 18]. Considering that few studies have focused on how FTO promotes tumour formation by regulating miRNA synthesis, our study demonstrated that FTO could promote bladder cancer proliferation via the FTO/miR-576/CDK6 pathway by regulating the maturation of primiR-576 in an m6A-dependent manner.

In the present study, the expression of FTO increased in bladder cancer tissues, which was consistent with the results of a study by Tao [9]. However, Wen et al. suggested that FTO is downregulated in bladder cancer [19], which is contrary to our conclusion. These contradictory results may be attributed to the sources the specimens were obtained from as well as the treatment they were subjected to after resection.

miRNAs play an important role in regulating gene expression at the post-transcriptional level by binding to the 3′-UTR of target mRNAs, thereby leading to target mRNA degradation or blocking their translation into proteins [20]. miR-576 is involved in the progression of many cancers, such as oesophageal cancer [21, 22], melanoma [23] and colorectal cancer [24]. In our study, we found that FTO regulates the processing of miR-576 in an m6A-depend manner.

Cyclin-dependent kinase 6 (CDK6), a serine/threonine-protein kinase, is a member of the cyclin-dependent kinase family. It is involved in G1 progression and the G1/S transition and accelerates progression through the G1/S-phase checkpoint of the cell cycle [25, 26]. CDK6 overexpression increases cell proliferation and reduces DNA repair capacity [26]. An increasing number of studies have shown that the aberrant expression of CDK6 is closely associated with the occurrence and development of many cancers, including breast cancer [27], ovarian cancer [28], colon cancer [29], bladder cancer [30, 31] and more [32, 33]. Studies have shown that CDK6 promotes cell proliferation in bladder cancer as it does in other tumour types [30, 31]. Zhao et al. reported that CDK6 was overexpressed in bladder cancer tissues [34]. In addition, in our cell cycle analysis, the cell cycle was arrested in the G0/G1 stage after FTO knockdown, which might be explained by the corresponding decreased expression of CDK6 in bladder cancer cells.

**Fig. 8:**
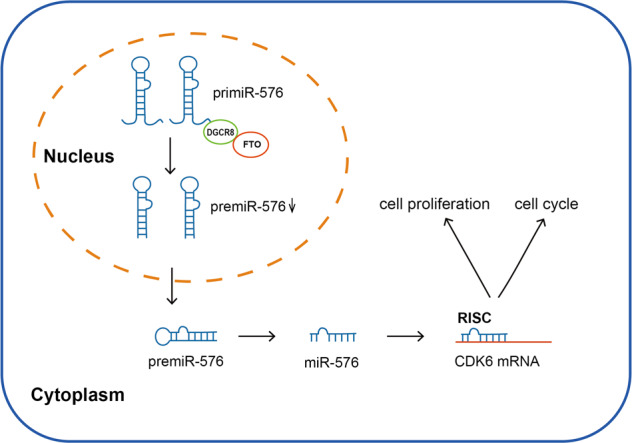
Mode pattern. FTO promotes tumour proliferation via the FTO/miR-576/CDK6 axis in an m6A-dependent manner.

In summary, our research verified that FTO was highly expressed in bladder cancer and associated with a poor prognosis in bladder cancer patients. FTO promoted bladder cancer cell proliferation, migration and invasion via the FTO/miR-576/CDK6 pathways in an m6A-dependent manner (Fig. 8). Our work revealed that FTO plays a critical role in bladder cancer and could be a potential diagnostic or prognostic biomarker for this disease.

## Materials and methods

### Patients and tissue samples

Twenty fresh bladder cancer tissue samples and their corresponding adjacent normal tissues (ANTs) were obtained from bladder cancer patients who underwent radical cystectomy surgery at Qilu Hospital of Shandong University between 2018 and 2020. This study was approved by the institutional review board of Qilu Hospital (No.2020046).

### Cell culture

The three bladder cancer cell lines (T24, 5637 and UM-UC-3) were purchased from the Chinese Academy of Science Committee Type Culture Collection Cell Bank (Shanghai, China). T24 and 5637 cells were cultured in RPMI 1640 medium (Gibco, USA) containing 10% foetal bovine serum (FBS) (Biological Industries, Israel). UM-UC-3 cells were cultured in a DMEM medium (Gibco, USA) containing 10% foetal bovine serum (FBS) (Biological Industries, Israel). The cell lines were cultured in a humidified atmosphere of 5% CO_2_ at 37 °C.

### Cell transfection and stable cell line construction

The lentivirus constructs of FTO overexpression or knockdown were purchased from Genechem (Shanghai, China). Briefly, T24, 5637 and UM-UC-3 were plated in six-well dishes and infected with FTO-overexpression lentivirus, negative control, FTO knockdown lentivirus and scramble control at 40% confluence. The viral titre was determined according to the manufacturer’s instructions. Seventy-two hours after transfection, puromycin (2 μg/mL) was used to select stable transductions.

The miRNA mimics or inhibitors were purchased from GenePharma (Shanghai, China) and were transfected into the cells using Lipofectamine 3000 reagent (Invitrogen, USA).

All lentivirus constructs, mimics, negative control, and inhibitor sequences are listed in Supplementary Table 2.

### Tissue microarray (TMA) and immunohistochemistry (IHC)

A TMA kit was purchased from Outdo Biotech Company (Shanghai, China) and contained 40 cases of paired bladder cancer tissues and 28 cases of unpaired bladder cancer tissues. All tissues were formalin-fixed and paraffin-embedded. The bladder cancer tissues, adjacent normal tissues and xenograft tumour tissues were collected in formaldehyde, embedded in paraffin and cut into 4-μm-thick sections. After antigen retrieval, each section was incubated with anti-FTO antibody (1:200, Abcam, USA), anti-CDK6 antibody (1:200, Abcam, USA), and anti-Ki-67 antibody (1:200, Bioss, China) at 4 °C overnight. The sections were then incubated with secondary antibody (1:1 000, Boster, China) for 1 h at room temperature. Images were acquired using a microscope (Aoren, China).

### RNA isolation and quantitative RT-PCR

TRIzol reagent (Invitrogen, USA) was used to isolate total RNA from tissues and cells. The Biospin miRNA Extraction Kit (Bioer Technology, China) was used to extract miRNAs according to the manufacturer’s instructions. Evo M-MLV RT Premix (Accurate Biology, China) and Mir-X™ miRNA First-Strand Synthesis (Takara, USA) were used to synthesise cDNA from total RNA and miRNA, respectively. qRT-PCR was performed using the Premix Pro Taq HS qPCR Kit (Accurate Biology, China) on a LightCycler 480 (Roche, USA) or ABI QuantStudio 3 system (Thermo, USA). GAPDH and U6 were served as internal standard controls. All assays were replicated three times. The primers used for qRT-PCR were purchased from Accurate Biology (China). The sequences of all primers are listed in Supplementary Table 3.

### Western blotting

Total proteins were isolated from tissues and cells using RIPA lysis buffer mixed with phenylmethylsulfuryl fluoride (PMSF) (Bioss, China) and quantified using the bicinchoninic acid (BCA) method (Beyotime, China). Equal amounts of proteins were separated by 10% SDS-PAGE and transferred onto PVDF membranes (Millipore, USA). After blocking by 5% skim milk, the PVDF membranes were incubated with an anti-FTO antibody (1:1 000, Abcam, USA), anti-CDK6 antibody (1:1 000, Abcam, USA) and anti-GAPDH antibody (1:5 000, Bioss, China) at 4 °C overnight. The PVDF membranes were then incubated with a secondary antibody (1:10 000, Boster, China) for 1 h at room temperature. The proteins were measured using the AMERSHAM ImageQuant 800 system (USA).

### RNA m6A quantification

TRIzol reagent (Invitrogen, USA) was used to isolate total RNA from cells. The relative m6A content was measured using the EpiQuik m6A RNA Methylation Quantification kit (colorimetric) (EpiGentek, USA) according to the manufacturer’s instructions. The percentage of m6A in total RNA could be calculated using the following formula: m6A% = [(sample OD − NC OD)/S]/[(PC OD − NC OD)/P] × 100%, where the NC (negative control) was an RNA containing no m6A, PC (positive control) stood for m6A oligos and was normalised to have 100% of m6A, S was the amount of input sample RNA and P was the amount of input positive control.

### Cell counting kit-8 (CCK-8), transwell migration and invasion assays

For the CCK-8 assay, transfected cells were seeded into 96-well plates (2000 cells per well). The Cell Counting kit-8 (CCK-8) (Bioss, China) was used to detect cell proliferation at 0, 24, 48, 72 and 96 h after culture. The absorbance was detected at 450 nm using a spectrophotometer (Tecan, Switzerland).

The transwell assay was performed using an 8.0 Corning™ 24-well Transwell assay plate (Corning, USA) according to the manufacturer’s instructions. After 24 h at 37 °C in an incubator at 5% CO_2_, the cells below the membrane were fixed with methanol and stained with crystal violet. The cell numbers in the three random fields were counted.

### Colony formation assay

The stably transfected cells were seeded into six-well plates (800 cells per well). After 2 weeks at 37 °C in an incubator at 5% CO_2_, the colonies were fixed with 100% methanol and stained with crystal violet. Colonies containing more than 50 cells were counted as survivors.

### Cell cycle distribution analysis

Transfected cells were fixed with 75% cold ethanol at −20 °C for 24 h. Then, 5 μL of propidium iodide (PI) (KeyGen BioTECH, China) was added to cell suspensions. The cell cycle of the cell suspension was analysed using the FACS Calibur Flow Cytometer (BD Biosciences, USA).

### RNA immunoprecipitation (RIP) assay

The Magna RIP RNA-Binding Protein Immunoprecipitation Kit (Millipore, USA) was used for the RIP assay, according to the manufacturer’s instructions. Briefly, stably transfected T24 cells were harvested and lysed in RIP lysis buffer. The RIP lysate was then incubated with magnetic beads conjugated with anti-DGCR8 antibody (Abcam, USA), anti-m6A antibody (Cell Signaling Technology, USA) or anti-immunoglobulin G (IgG, Millipore, USA) antibody at 4 °C overnight. Then, the immunoprecipitated RNAs were extracted using TRIzol reagent (Invitrogen, USA) and subjected to qRT-PCR using primiR-576 primers.

### Dual-luciferase reporter assay

To verify the target gene of miR-576, a dual-luciferase reporter assay was performed. The pmirGLO luciferase vector (GeneCreat, China) was constructed with target sequences containing the predicted miR-576 binding sites. The cells were then co-transfected with the luciferase vector and miR-576 mimics or its control (mimic-NC) using Lipofectamine 3000 (Invitrogen, USA). After being incubated for 24 h, firefly and Renilla luciferase activities were detected using the Dual-Luciferase Reporter Assay System (Promega, USA).

### In vivo experiments

All animal studies were performed in accordance with the Guide for the Care and Use of Laboratory Animals and approved by the Animal Care Committee of Shandong University. BALB/c nude mice (female, 6 weeks old, five mice per group) were purchased from Vital River Laboratories (Beijing, China). Approximately 1 × 10^7^ stably transfected T24 cells were subcutaneously injected into BALB/c nude mice. The length (L) and width (W) of the tumours were measured weekly using callipers, while their volume was calculated using the equation: V = (L × W^2^)/2. After 4 weeks of injections, the mice were euthanised, and the tumour tissues were removed and weighed. Some tumour tissues were used to obtain total RNA and protein data, and the rest were fixed with methanol and embedded in paraffin for IHC.

### Statistical analysis

Data analysis was performed using the SPSS version 22.0. The results are presented as mean ± standard deviation (SD). The relationship between FTO expression and clinicopathological features was analysed using the chi-square test. Between-group differences were analysed using Student’s *t*-test. Overall survival (OS) was evaluated using the Kaplan–Meier method. The significance level was set at *P* < 0.05 and each experiment was performed in triplicate.

## Supplementary information

Supplementary Figure Legends

Supplementary figure 1

Supplementary figure 2

Supplementary table 1

Supplementary table 2

Supplementary table 3

## Data Availability

The data that support the findings of this study are available from the corresponding author upon reasonable request.
