# Correction: FTO promotes tumour proliferation in bladder cancer via the FTO/miR-576/CDK6 axis in an m6A-dependent manner

**DOI:** 10.1038/s41420-021-00762-z

**Published:** 2021-12-02

**Authors:** Guanwen Zhou, Keqiang Yan, Jikai Liu, Lijian Gao, Xianzhou Jiang, Yidong Fan

**Affiliations:** 1 Department of Urology, Qilu Hospital, Cheeloo College of Medicine, Shandong University, Jinan, China; 2 Department of Urology, Dezhou People’s Hospital, Dezhou, China

**Keywords:** miRNAs, Bladder cancer, Urological cancer, Methylation

Correction to: *Cell Death Discovery* 10.1038/s41420-021-00724-5 published online 1 November 2021

The original version of this article unfortunately contained a mistake in Table 1. In TNM stage, the number of cases of two groups should be 21 (pTa–pT1) and 46(pT2–pT4). This mistake has no impact on the research results. The authors apologize for the mistake. The corrected table can be found below. The original article has been corrected.

**Table 1:** Correlation between FTO expression and clinicopathological characteristics of 67 bladder cancer patients.

Parameters	Number of cases	FTO expression^a^		*P* value
		Low	High	
**All cases**	67	23	44	
**Age (years)**				
<65	24	8	16	0.995
≥65	43	15	28	
**Gender**				
Male	57	20	37	0.755
Female	10	3	7	
**TNM stage**				
pTa–pT1	21	11	10	0.035^b^
pT2–pT4	46	12	34	
**Histological grade**				
Low	9	5	4	0.14
High	58	18	40	

^a^On the basis of the IHC scores, the patients were divided in.to low (0–3) and high (4–9) FTO groups. Final IHC score = percentage of positive cells × intensity score.

^b^Statistically significant, *P* < 0.05.

