# Supplementary material for: FTO promotes tumour proliferation in bladder cancer via the FTO/miR-576/CDK6 axis in an m6A-dependent manner
Source: Cell Death Discov. 2021 Nov 1;7:329. doi: 10.1038/s41420-021-00724-5 (PMC8560827; doi:10.1038/s41420-021-00724-5)
Supplement: Supplementary file 1 — Supplementary Figure Legends [file 41420_2021_724_MOESM1_ESM.docx]

**Supplementary Figure 1.**  **The overexpression of FTO promoted cell proliferation and invasion in 5637 cell lines. A.** FTO was detected by western blotting after FTO overexpression. **B**. The m6A levels were measured in FTO-overexpressing 5637 cells. **C**. FTO overexpression promoted cell proliferation determined by CCK-8 assays. **D**. Transwell migration and invasion assays after FTO overexpression in 5637 cells at a 100× magnification. Scale bars represent 100 μm. **E**. Cell-cycle analysis was performed in FTO-overexpressing 5637 cells using flow cytometry. The results are presented as mean ± standard deviation (SD). *P < 0.05, **P< 0.01, ***P < 0.001.

**Supplementary Figure 2. Analysis of miRNAs negatively correlated with FTO using the LinkedOmics database.**
