# Supplementary figures and images for: FTO promotes tumour proliferation in bladder cancer via the FTO/miR-576/CDK6 axis in an m6A-dependent manner
Source: Cell Death Discov. 2021 Nov 1;7:329. doi: 10.1038/s41420-021-00724-5 (PMC8560827; doi:10.1038/s41420-021-00724-5)

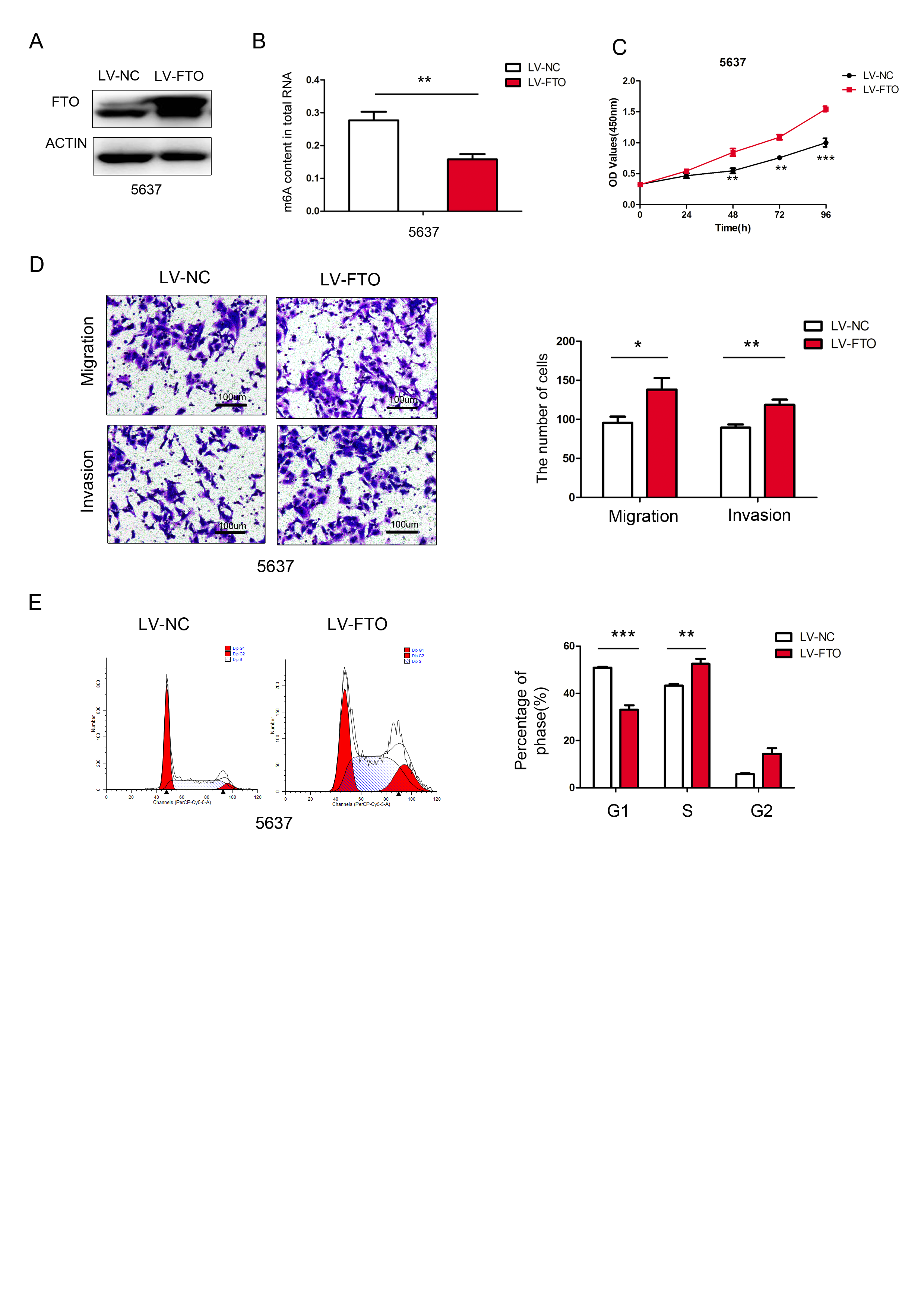

Supplement: Supplementary file 2 — Supplementary figure 1 [file 41420_2021_724_MOESM2_ESM.png]

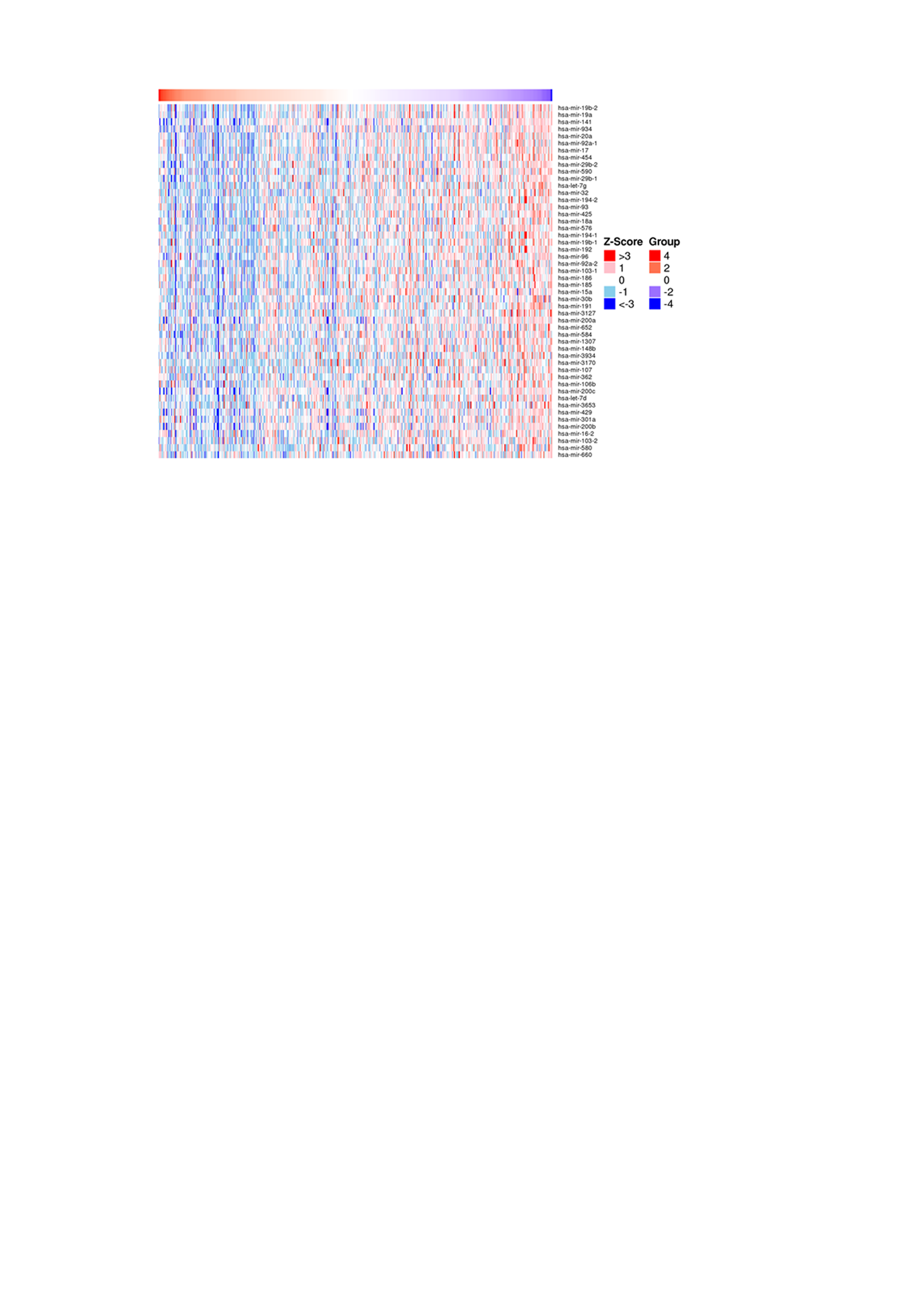

Supplement: Supplementary file 3 — Supplementary figure 2 [file 41420_2021_724_MOESM3_ESM.png]
