# Supplementary material for: FTO promotes tumour proliferation in bladder cancer via the FTO/miR-576/CDK6 axis in an m6A-dependent manner
Source: Cell Death Discov. 2021 Nov 1;7:329. doi: 10.1038/s41420-021-00724-5 (PMC8560827; doi:10.1038/s41420-021-00724-5)
Supplement: Supplementary file 5 — Supplementary table 2 [file 41420_2021_724_MOESM5_ESM.docx]

**Supplementary Table 2.** Lentivirus constructs, mimics, and inhibitor sequences used in this study.

| Gene | Sequences (5’-3’) |
| --- | --- |
| shFTO | gcAGCATACAACGTAACTTTG |
| miR-576 mimics | sense: AUUCUAAUUUCUCCACGUCUUU |
|  | antisense: AGACGUGGAGAAAUUAGAAUUU |
| miR-576 NC | sense: UUCUCCGAACGUGUCACGUTT |
|  | antisense: ACGUGACACGUUCGGAGAATT |
| miR-576 inhibitors | AAAGACGUGGAGAAAUUAGAAU |
| miR-576 inhibitors NC | CAGUACUUUUGUGUAGUACAA |
