# Supplementary material for: FTO promotes tumour proliferation in bladder cancer via the FTO/miR-576/CDK6 axis in an m6A-dependent manner
Source: Cell Death Discov. 2021 Nov 1;7:329. doi: 10.1038/s41420-021-00724-5 (PMC8560827; doi:10.1038/s41420-021-00724-5)
Supplement: Supplementary file 6 — Supplementary table 3 [file 41420_2021_724_MOESM6_ESM.docx]

**Supplementary Table 3.** Primes sequences for qRT-PCR used in this study.

| **Gene** | **Sequences (5’-3’)** |
| --- | --- |
| FTO-forward | GAAGCACTGTGGAAGAAGATGGA |
| FTO-reverse | GGCAAGGATGGCAGTCAAGAT |
| miR-576-forward | GGCTAATTTCTCCACGTCTTT |
| pri-miR-576-forward | AGAATTGCTGGGTCAGAACTCT |
| pri-miR-576-reverse | TGCTCAACATCACAATCCCATTAG |
| pre-miR-576-forward | CCAACGAGGATTCTAATTTCTCC |
| pre-miR-576-reverse | ACCAATCGAATGAGGATTCCA |
| CDK6-forward | GTGACCAGCAGCGGACAAATAA |
| CDK6-reverse | AGCAAGACTTCGGGTGCTCTGTA |
| GAPDH-forward | GCACCGTCAAGGCTGAGAAC |
| GAPDH-reverse | TGGTGAAGACGCCAGTGGA |
| U6-forward | GGAACGATACAGAGAAGATTAGC |
| U6-reverse | TGGAACGCTTCACGAATTTGCG |
